# Web-based Surveillance and Global *Salmonella* Distribution, 2000–2002

**DOI:** 10.3201/eid1203.050854

**Published:** 2006-03

**Authors:** Eleni Galanis, Danilo M.A. Lo Fo Wong, Mary E. Patrick, Norma Binsztein, Anna Cieslik, Thongchai Chalermchaikit, Awa Aidara-Kane, Andrea Ellis, Frederick J. Angulo, Henrik C. Wegener, for World Health Organization Global Salm-Surv

**Affiliations:** * Danish Institute for Food and Veterinary Research, Søborg, Denmark;; † Instituto Nacional de Enfermedades Infecciosas ANLIS “Carlos G. Malbran,” Buenos Aires, Argentina;; ‡ National Institute of Hygiene, Warsaw, Poland;; § Chulalongkorn University, Bangkok, Thailand;; ¶ World Health Organization, Geneva, Switzerland;; # Institut Pasteur, Dakar, Senegal; ** Public Health Agency of Canada, Guelph, Ontario, Canada;; †† Centers for Disease Control and Prevention, Atlanta, Georgia, USA

**Keywords:** Salmonella, epidemiology, world health

## Abstract

Salmonellae are a common cause of foodborne disease worldwide. The World Health Organization (WHO) supports international foodborne disease surveillance through WHO Global Salm-Surv and other activities. WHO Global Salm-Surv members annually report the 15 most frequently isolated *Salmonella* serotypes to a Web-based country databank. We describe the global distribution of reported *Salmonella* serotypes from human and nonhuman sources from 2000 to 2002. Among human isolates, *S*. Enteritidis was the most common serotype, accounting for 65% of all isolates. Among nonhuman isolates, although no serotype predominated, *Salmonella enterica* serovar Typhimurium was reported most frequently. Several serotypes were reported from only 1 region of the world. The WHO Global Salm-Surv country databank is a valuable public health resource; it is a publicly accessible, Web-based tool that can be used by health professionals to explore hypotheses related to the sources and distribution of salmonellae worldwide.

## Abstract 2

Surveillance improves control of *Salmonella* infections.

Foodborne diseases are among the most serious health problems affecting public health and development worldwide (*1*). Industrialization, mass food production, decreasing trade barriers, and human migration have disseminated and increased the incidence and severity of foodborne diseases worldwide (*2**–**4*).

Salmonellae are among the most common bacterial foodborne pathogens worldwide (*4*). They cause an estimated 1.4 million cases of foodborne disease each year in the United States alone (*5*). *Salmonella* serotyping is a surveillance tool that detects widespread outbreaks, identifies outbreak sources, monitors trends over time, and attributes human disease to various foods and animals (*6*). Such surveillance is needed to help prevent foodborne disease outbreaks and raise awareness among health authorities, food producers, food regulators, and consumers (*7*).

A 1997 survey of national reference laboratories showed that only 69 (66%) of 104 responding countries conducted routine *Salmonella* serotyping for public health surveillance (*8*). Consequently, the World Health Organization (WHO), the US Centers for Disease Control and Prevention, and the Danish Veterinary Laboratory (now the Danish Institute for Food and Veterinary Research) founded WHO Global Salm-Surv in 2000. Its mission is to promote integrated, laboratory-based surveillance and foster collaboration among human health, veterinary, and food-related disciplines to enhance the capacity to detect, respond, and prevent foodborne diseases (*9*). By November 2005, WHO Global Salm-Surv had >800 members from 142 countries. A key component of this program is the Web-based country databank, to which member countries annually submit their 15 most frequently isolated *Salmonella* serotypes. This program is the only foodborne disease surveillance network that is global in scope and surveys all aspects of the food chain, from animal feed to humans. Data are updated annually and are publicly accessible for members and the scientific community to review (http://www.who.int/salmsurv). We describe the global distribution of *Salmonella* serotypes from human and nonhuman sources reported to the WHO Global Salm-Surv country databank from 2000 to 2002 and explore how the databank may become a valuable public health resource for foodborne disease surveillance.

## Methods

WHO Global Salm-Surv has conducted annual regional training courses for national reference laboratories since 1999 and has managed an external laboratory quality assurance program since 2000 to facilitate a standard approach to isolating and serotyping salmonellae (*10*). National reference laboratories can become WHO Global Salm-Surv members and share *Salmonella* serotype data with other members through the country databank. The country databank is a Web-enabled Oracle database that is password protected for data entry and accessible for public viewing at http://www.who.int/salmsurv. Each year, a designated national reference laboratory representative enters into the country databank the number of *Salmonella* isolates serotyped from human, animal, food, environmental, and feed sources and the 15 most frequently identified serotypes.

Descriptive analysis was conducted by using Microsoft Excel (Microsoft Corp., Redmond, WA, USA) on data from all countries that submitted data for 2000, 2001, or 2002 as of June 2004. Analyses for trends over time were conducted on data from 2000 to 2002. More detailed analyses, including ranking of serotypes, comparison of human to nonhuman isolates, and regional comparisons are presented for 2002 data only, the year in which the most countries participated.

Before 2001, nonhuman isolates were grouped together. Since 2001, countries have been able to submit food, animal, environmental, and feed data separately. For comparison purposes, all nonhuman data were combined in this analysis.

Data were grouped into regions approximately corresponding to 6 geopolitical continents: Africa, Asia, Latin America and the Caribbean, Europe, North America, and Oceania. To accommodate local epidemiologic characteristics, New Caledonia was incorporated into Asia, and Israel was incorporated into Europe. For years in which a single country contributed data for a region, regional data are not presented, but the data are included in the overall results. A region-specific serotype was defined as a serotype that, for each of the years of the study period, was among the 15 most commonly reported serotypes and for which >90% of the isolates were from that region.

## Results

### Global

Forty-nine countries submitted data to the WHO Global Salm-Surv country databank from 2000 to 2002 (Table 1). Twenty countries reported both human and nonhuman results, 21 reported only human results, and 8 reported only nonhuman results. Reports of 376,856 human and 65,789 nonhuman *Salmonella* isolations were entered into the database during the 3-year period. North America and Europe accounted for 87.9% (389,134) of all reported isolates. The number of isolates reported to the country databank was stable during the study period; 113,782–137,329 human isolates and 16,506–25,761 nonhuman isolates were reported per year.

**Table 1:** Number of serotyped *Salmonella* isolates reported to the World Health Organization Global Salm-Surv country databank, 2000–2002

Country	Human			Nonhuman		
	2000	2001	2002	2000	2001	2002
Africa	104	406	965	33	101	1,477
Cameroon		263	247		12	10
Mali			34			
Morocco			76			
Senegal	104	143	220	33	89	91
Tunisia			388			1,376
Asia	8,233	6,696	5,771	4,056	1,513	1,631
China				43	98	127
Indonesia				219		
Japan	2,631	2,452	1,890			
Korea	1,260	918	843			
Malaysia	499			1,390		
New Caledonia		30	20			
Philippines	606					
Thailand	3,233	3,279	2,922	2,404	1,415	1,504
Vietnam	4	17	96			
Europe	91,788	73,556	85,385	10,628	8,951	3,113
Belgium	13,642	10,260				
Bulgaria	789	1,001	1,482			
Cyprus				52		45
Czech Republic	4,774	4,030	27,381			
Denmark	2,063	2,632	1,844	5,981	5,402	
Estonia				178	38	78
Germany				3,068		
Greece				337		842
Hungary	16,271	14,462	14,678	748	1,448	1,809
Israel	4,428	4,043	3,859			
Latvia					139	132
Luxembourg	381					
Norway	1,289	1,639				
Poland	38,138	26,601	28,705	234	524	151
Portugal	354	539				
Serbia and Montenegro	5,172	5,003	4,873		46	
Slovakia				30	1,354	56
Slovenia	3,456	1,576	2,563			
Switzerland	1,031	1,770				
Latin America and Caribbean	2,054	2,239	2,491	411	633	727
Argentina	633	608	487	124	165	147
Barbados		27	71		24	23
Bolivia		19	9		8	2
Chile	929	920	1,284	218	329	395
Colombia	145	135	194		31	52
Costa Rica		49			11	49
Cuba			65			
El Salvador			149			
Peru	115	120	49	7	5	19
Suriname			18			
Trinidad		67				
Venezuela	232	294	165	62	60	40
North America	29,201	28,508	29,301	8,808	10,337	9,558
Canada	4,788	4,992	4,962	3,588	4,743	4,676
USA	24,413	23,516	24,339	5,220	5,594	4,882
Oceania	5,949	2,377	1,832	1,825	1,987	
Australia	4,202					
New Zealand	1,747	2,377	1,832	1,825	1,987	
Total countries	29	31	31	20	22	22
Total isolates serotyped	137,329	113,782	125,745	25,761	23,522	16,506

**Figure 1:**
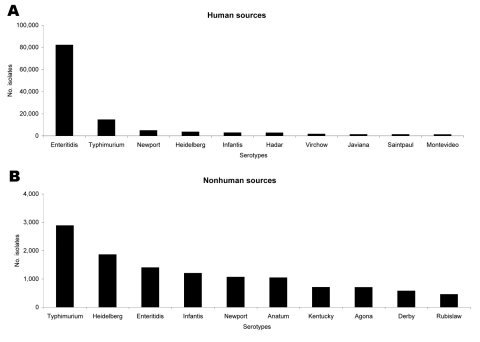
Number of *Salmonella* isolates reported by serotype worldwide in 2002. A) Human sources; B) nonhuman sources.

During the 3-year period, *Salmonella enterica* serovar Enteritidis was by far the most common serotype reported from human isolates globally. In 2002, it accounted for 65% of all isolates, followed by *S*. Typhimurium at 12% and *S*. Newport at 4%. Among nonhuman isolates, *S*. Typhimurium was the most commonly reported serotype in all 3 years, accounting for 17% of isolates in 2002. It was followed by *S*. Heidelberg (11%) and *S*. Enteritidis (9%) (Figure 1).

In 2002, 26 (84%) of the 31 countries that reported human serotype results ranked *S*. Enteritidis and *S*. Typhimurium in their 10 most common human serotypes (Table 2). Approximately half of the countries ranked *S*. Infantis and *S*. Typhi in their 10 most common serotypes, but only a fourth ranked *S*. Newport and *S*. Heidelberg in their top 10. The relative ranking of serotypes by the number of countries reporting them in their 15 most frequent serotypes remained stable over the study period (data not shown). However, the proportion of countries reporting each serotype varied. For example, from 2000 to 2001, more than two thirds of countries reported *S*. Agona, compared to 39% in 2002.

**Table 2:** Number and proportion of countries (N = 31) that ranked in the top 10 each of the 20 most common *Salmonella* serotypes among human isolates, 2002

Global rank	Serotype	Europe, n (%)	Asia, n (%)	Oceania, n (%)	Africa, n (%)	North America, n (%)	Latin America and Caribbean, n (%)	Total, n (%)
1	Enteritidis	8 (100)	4 (80)	1 (100)	4 (80)	2 (100)	7 (70)	26 (84)
2	Typhimurium	8 (100)	5 (100)	1 (100)	4 (80)	2 (100)	6 (60)	26 (84)
3	Newport	3 (38)	1 (20)	0	1 (20)	2 (100)	1 (10)	8 (26)
4	Heidelberg	2 (25)	2 (40)	0	0	2 (100)	2 (20)	8 (26)
5	Infantis	8 (100)	1 (20)	1 (100)	1 (20)	2 (100)	1 (10)	14 (45)
6	Hadar	6 (75)	3 (60)	0	3 (60)	2 (100)	0	14 (45)
7	Virchow	5 (63)	0	1 (100)	2 (40)	0	1 (10)	9 (29)
8	Javiana	0	0	0	0	1 (50)	2 (20)	3 (10)
9	Saintpaul	3 (38)	1 (20)	1 (100)	0	2 (100)	3 (30)	10 (32)
10	Montevideo	2 (25)	2 (40)	1 (100)	1 (20)	2 (100)	4 (40)	12 (39)
11	Agona	6 (75)	1 (20)	0	0	2 (100)	3 (30)	12 (39)
12	Oranienburg	0	0	0	0	2 (100)	1 (10)	3 (10)
13	Thompson	3 (38)	1 (20)	1 (100)	0	2 (100)	0	7 (23)
14	Typhi	1 (13)	2 (40)	1 (100)	4 (80)	1 (50)	5 (50)	14 (45)
15	Muenchen	0	0	0	0	1 (50)	0	1 (3)
16	Paratyphi B d-tartrate+	2 (25)	0	0	0	2 (100)	0	4 (13)
17	Braenderup	0	1 (20)	0	1 (20)	0	2 (20)	4 (13)
18	Blockley	2 (25)	0	0	0	0	0	2 (6)
19	Anatum	1 (13)	1 (20)	0	0	0	3 (30)	5 (16)
20	Weltevreden	0	2 (40)	0	0	0	1 (10)	3 (10)

In 2002, a total of 5 serotypes were reported among the 15 most common human serotypes from all 6 regions of the world: *S*. Enteritidis, *S*. Typhimurium, *S*. Infantis, *S*. Montevideo, and *S*. Typhi. However, the proportion of isolates of each serotype varied greatly. In 2002, for example, *S*. Enteritidis represented 85% of isolates in Europe but only 9% in Oceania. In Latin America and the Caribbean, *S*. Typhi accounted for the greatest proportion of salmonellae (13%). In 2000 and 2001, *S*. Enteritidis, *S*. Typhimurium, *S*. Typhi, and *S*. Agona were reported from all 6 regions (data not shown).

*S*. Enteritidis, *S*. Typhimurium, and *S*. Typhi were ranked among the 15 most common human serotypes in all 6 regions throughout the 3-year study period. *S*. Agona, *S*. Infantis, *S*. Montevideo, *S*. Saintpaul, *S*. Hadar, *S*. Mbandaka, *S*. Newport, *S*. Thompson, *S*. Heidelberg, and *S*. Virchow were also widespread; they were reported from 4 to 6 of the regions from 2000 through 2002. Reporting of *S*. Montevideo increased from 4 regions in 2000 to all 6 regions in 2002. The reporting of *S*. Heidelberg increased from 3 to 5 regions in the same timeframe.

### Regional

**Figure 2:**
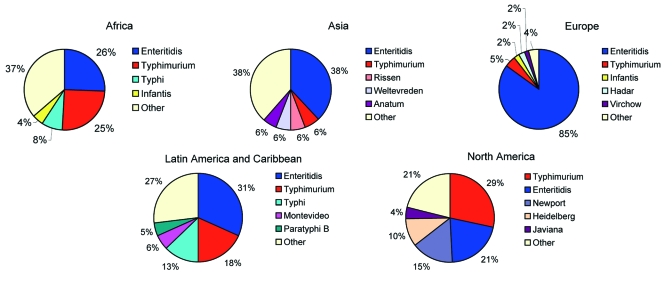
Proportion of most common serotypes of reported human *Salmonella* isolates by region, 2002.

**Figure 3:**
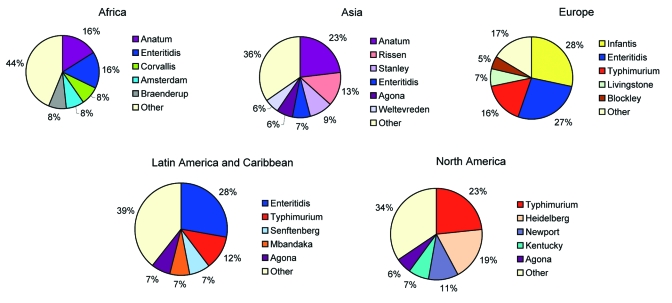
Proportion of most common serotypes of reported nonhuman *Salmonella* isolates by region, 2002.

In Africa in 2002, *S*. Enteritidis and *S*. Typhimurium were each reported from approximately one fourth of isolates from humans (Figure 2). Among nonhuman sources (Figure 3), *S*. Anatum and *S*. Enteritidis constituted the largest proportion of isolates.

In Asia, from 2000 through 2002, Japan, Korea, and Thailand together reported *S*. Enteritidis as the most common human serotype. *S*. Weltevreden was the second most common serotype in 2000 and 2001 but dropped to fourth in 2002, when it was surpassed by *S*. Rissen and *S*. Typhimurium. In 2002, *S*. Enteritidis accounted for 38% of human isolates but only 7% of nonhuman isolates. *S*. Anatum, *S*. Rissen, and *S*. Stanley were the most common nonhuman serotypes in Asia.

In Europe in 2002, *S*. Enteritidis accounted for most salmonellae among human isolates. This trend was constant from 2000 to 2002; *S*. Enteritidis accounted for 79% to 84% of isolates, followed by *S*. Typhimurium in second place and *S*. Hadar, *S*. Virchow, and *S*. Infantis alternating in the third to fifth places among the 8 countries that submitted data during the 3 years. Among nonhuman isolates, heterogeneity was greater; *S*. Infantis, *S*. Enteritidis, and *S*. Typhimurium together accounted for 72% of salmonellae in 2002.

In 2002 in Latin America and the Caribbean, *S*. Enteritidis was the most common serotype among human and nonhuman isolates. *S*. Typhimurium, *S*. Typhi, *S*. Montevideo, and *S*. Paratyphi B were also commonly observed among human isolates and *S*. Typhimurium, *S*. Senftenberg, *S*. Mbandaka, and *S*. Agona, among nonhuman isolates. During the 3-year period of interest, *S*. Enteritidis, *S*. Typhimurium, and *S*. Typhi were the 3 most commonly isolated serotypes among humans in the 5 countries that reported data every year.

In North America in 2002, *S*. Typhimurium was more common than *S*. Enteritidis among human isolates. *S*. Newport and *S*. Heidelberg also accounted for a sizeable proportion of the isolates. Among nonhuman isolates, a corresponding pattern emerges; *S*. Typhimurium, *S*. Heidelberg, and *S*. Newport were most common. *S*. Enteritidis was not reported among the 10 most common nonhuman serotypes. The relative ranking of serotypes did not change in the 3-year period; *S*. Typhimurium was the most common serotype in humans and nonhuman isolates from 2000 to 2002. In Oceania in 2000, the only year in which >1 country reported data, *S*. Typhimurium accounted for 62% of human *Salmonella* isolates, followed by *S*. Virchow and *S*. Enteritidis.

Some serotypes were reported among the 15 most common serotypes in only 1 region during the 3-year period and therefore were classified as region-specific serotypes. Africa was the only region to report *S*. Brancaster among nonhuman isolates. Asia was the only region to report *S*. Rissen (human), *S*. Panama and *S*. Stanley (nonhuman), and *S*. Weltevreden (human and nonhuman). Europe was the only region to report *S*. Blockley, *S*. Kisangani, *S*. Kottbus, *S*. Ohio, and *S*. Stanleyville from human isolates and *S*. Indiana and *S*. Isangi from nonhuman isolates. Latin America and the Caribbean was the only region to report *S*. Bardo, *S*. Muenster, and *S*. Rubislaw among human isolates. North America was the only region to report *S*. Javiana (human) and *S*. Muenster (nonhuman).

## Discussion

*S*. Enteritidis is the most common *Salmonella* serotype in humans globally but especially in Europe, where it accounts for 85% of *Salmonella* cases, Asia (38%), and Latin America and the Caribbean (31%). The *S*. Enteritidis pandemic was first noted in the late 1980s and has been attributed to contaminated eggs (*11*). The proportion of *Salmonella* infections associated with this serotype seems to have increased over time. In 1995, 36% of salmonellae worldwide were *S*. Enteritidis, compared to 65% in 2002 (*8*).

*S*. Typhimurium has been 1 of the 2 most frequent serotypes in humans since 1990 (*8*). Since *S*. Enteritidis and *S*. Typhimurium are so common, additional subtyping methods, including phage typing, antimicrobial susceptibility testing, and pulsed-field gel electrophoresis (PFGE), are needed to identify clusters of infection from the same source. WHO Global Salm-Surv includes antimicrobial susceptibility testing training in all regional courses and has introduced phage typing in the Eastern European region course. The country databank could include data from such subtyping efforts. PFGE subtyping data are exchanged in North American between PulseNet USA and PulseNet Canada (*12*). PulseNet International is an affiliate member of WHO Global Salm-Surv, and the networks are coordinating their efforts to ensure synergy.

*S*. Typhi is a pathogen of concern in the developing world, especially Asia (*13*). However, in our analyses, *S*. Typhi was the ninth most frequent serotype in Asia in 2002. The Asian countries that contributed to the country databank did not include many of the developing countries in south-central and Southeast Asia, where *S*. Typhi is still highly prevalent. *S*. Typhi was the sixth most frequent serotype globally in 1995 and was decreasing in relative importance (*8*). That trend seems to have continued; *S*. Typhi ranked 14th globally in 2002. *S*. Typhi has no animal reservoir, which makes it susceptible to improvements in hygiene and sanitation seen in many regions of the world, such as Latin America and the Caribbean.

The distribution of nonhuman serotypes is more heterogeneous than that of human serotypes. The same serotypes appear among the top 5 in human and nonhuman sources, although in a different order. *S*. Enteritidis is only the third most common serotype among nonhuman sources. In 2002, it was not reported at all among the 10 most common nonhuman serotypes from North America. This finding partly reflects the capacity of *S*. Enteritidis to contaminate eggs in low numbers and the difficulty of isolating it from food or the environment. Moreover, in North America, few samples from eggs are submitted for routine testing. For example, in the United States, routine testing of eggs is not required, whereas routine testing for salmonellae is required of meat and poultry plants. As eggs are frequently used in foods that do not undergo heat treatment (e.g., pastries, homemade ice cream, and mayonnaise) and are widely distributed, this food contamination has a substantial effect on public health.

The country databank contains far fewer nonhuman than human serotypes, possibly because more participating laboratories are human national reference laboratories, fewer countries have formal nonhuman surveillance, and some countries may be less likely to share nonhuman data because of trade concerns. In 2001 and 2002, 15 of 22 countries reported nonhuman isolates by source. Food serotypes were reported from most countries (11 in 2001 and 12 in 2002), followed by animal serotypes (7 countries in 2001 and 10 in 2002). Most isolates serotyped were from animals (66%), followed by food (29%), feed (3%), and the environment (2%). The reporting of *S*. Weltevreden from the environment, feed, animals, food, and humans in Southeast Asia is an example of how the country databank can be used to track *Salmonella* serotypes along the food chain.

Many serotypes are restricted to a single region of the world. This finding may reflect an ecologic niche or a local food source that is not exported. A number of such examples have been reported in the past, such as *S*. Marina associated with marine iguanas from South America found in the United States and *S*. Tilene in imported African pygmy hedgehogs in the United States and Canada (*14**–**16*). The country databank is uniquely placed to allow countries to observe this phenomenon. Investigators have reported infections of *S*. Javiana associated with exposure to wild amphibians in a confined area in the southeastern United States (*17*). According to the country databank, *S*. Javiana is only reported among the 15 most common serotypes in the United States. In 2001, the WHO Global Salm-Surv country databank helped confirm that *S*. Weltevreden was largely restricted to Southeast Asia. A survey of Southeast Asian laboratories showed that items most frequently associated with this serotype include seafood, water, and Asian vegetables (*18*). In the same region, *S*. Rissen has increased in both human and nonhuman sources (*19*). The country databank allows countries to become aware that a common serotype in their country may be rare elsewhere in the world, leading to hypothesis generation in outbreaks and studies to understand the sources of disease. Countries that report a large number of isolates to the country databank, such as North American and European countries, typically do not report rare serotypes because these would not rank in their top 15, thus limiting the ability to track rare serotypes in these countries.

Countries with fewer resources may lack complete antisera kits necessary to identify certain serotypes, which would lead to underreporting. For example, although we assume that *S*. Enteritidis human infections occur globally, a number of countries in Asia, Africa, and Latin America and the Caribbean did not report this serotype in their top 10 (Table 2). Lack of resources can also cause misclassification of serotypes. For example, *S*. Paratyphi B was reported to be among the most common serotypes in Latin America and the Caribbean. However, some countries in the region lack the capacity to differentiate between *S*. Paratyphi B and *S*. Paratyphi B tartrate var. Java.

In general, industrialized countries are more likely to regularly contribute to the country databank and to report more isolates. The results are therefore biased towards the industrialized world. However, the country databank lacks data from many Western European countries. Twenty-four European countries report human *Salmonella* serotype results annually to Enter-net, a European-based surveillance network for gastrointestinal infections, as compared to only 14 reporting to WHO Global Salm-Surv from 2000 to 2002. A review of recent Enter-net data confirms that *S*. Enteritidis is by far the most commonly isolated serotype in Europe but at a lower proportion than that reported to WHO Global Salm-Surv; 68%–71% of Enter-net *Salmonella* isolates were *S*. Enteritidis from 2000 to 2002 (Ian Fisher, pers. comm.) (*20*). A recent agreement between WHO Global Salm-Surv and Enter-net will lead to routine electronic sharing of data between the 2 systems to improve efficiency and representativeness (Henrik Wegener, pers. comm.).

Serotypes reported by a region are not necessarily circulating locally and may have been imported through travel or traded foods. Intraregional comparisons are limited by the fact that case definitions and surveillance systems vary between countries. The country databank does not collect the source of isolation. Some countries may report salmonellae isolated from both blood and stool and others from stool only. The low number of isolates and countries reporting nonhuman data and the pooling of food, animal, environmental, and feed sources hamper further analysis of nonhuman data. Some regional results may not be representative, since some regions have few countries reporting data to the country databank (e.g., Africa).

Some countries may not submit data to the country databank because of concern regarding international trading of food. Others do not have the supplies or training necessary to conduct serotyping. WHO Global Salm-Surv training courses were launched in Southeast Asia in 1999 and expanded to South America and the Middle East in 2000; China, Central America, and the Caribbean in 2001; and West Africa and Eastern Europe in 2002 (http://www.who.int/salmsurv). All participating countries were initially provided with antisera to conduct serotyping. WHO Global Salm-Surv established an external quality assurance system (EQAS) in 2000 to assess the accuracy of serotyping and antimicrobial susceptibility testing among member national reference laboratories. From 2000 to 2002, the number of laboratories participating in EQAS increased from 44 to 117, and the capacity to correctly serotype 8 *Salmonella* isolates improved from 76% to 90% of participating laboratories (*21*). The increased reporting of certain serotypes during the study period may be due to capacity improvement and increased participation in the country databank as well as real changes in the epidemiologic features of salmonellae. For example, *S*. Montevideo was first reported in Africa in 2001. In 2002, Tunisia participated in the country databank for the first time and reported *S*. Montevideo in its top 15 (increased participation). Oceania started reporting *S*. Montevideo in its top 15 in 2002 for the first time since participating in the country databank (change in epidemiologic features).

Data are not entered into the country databank in a timely enough way to detect international outbreaks. However, regularly monitoring the data can allow emerging trends in regional and international *Salmonella* epidemiology and region-specific serotypes to be detected. This information in turn leads to hypothesis generation, studies, and international collaboration to improve control of salmonellae in the long term. Examples of such work include the surveillance of *Salmonella* serotypes and antimicrobial drug resistance in South America, China, and the Democratic Republic of Congo; the assessment of risk factors for and drug resistance of *S*. Weltevreden in Southeast Asia; and the molecular characterization of *S*. Corvallis isolates from Bulgaria, Thailand, and Denmark (18,22–26, F. Aarestrup, pers. comm.).

The WHO Global Salm-Surv Country Databank is a valuable resource for international *Salmonella* surveillance. Past attempts to characterize *Salmonella* serotype distribution globally have either not been widely accessible or relied on irregular surveys of laboratories (*8**,**11*). Trends in global *Salmonella* epidemiology can now be updated and followed across regions and over time. In an era when most national institutions have access to the Internet, using a Web-based data collection tool is both feasible and practical. The data are immediately and publicly accessible for viewing and analysis (http://www.who.int/salmsurv). The results have several limitations in terms of representativeness and comparability but can be used to follow trends, generate hypotheses, and assess the effect of major interventions. This surveillance is a step toward improving the understanding and control of salmonellae worldwide.
